# A Novel Flow Cytometry Array for High Throughput Detection of SARS-CoV-2 Antibodies

**DOI:** 10.3390/vaccines13101063

**Published:** 2025-10-17

**Authors:** Benyue Zhang, Zhuo Zhang, Yichao Zhao, Jingqiao Lu, Jianmin Fang, Brianne Petritis, Kelly Whittaker, Rani Huang, Ruo-Pan Huang

**Affiliations:** 1 RayBiotech Life, Inc., Peachtree Corners, GA 30092, USA; ben.zhang@raybiotech.com (B.Z.);; 2 RayBiotech Guangzhou Co., Ltd., 79 Ruihe Road, Huangpu District, Guangzhou 510535, China; support_cn@raybiotech.cn; 3 South China Biochip Research Center, 79 Ruihe Road, Huangpu District, Guangzhou 510535, China

**Keywords:** COVID-19 antibodies, flow cytometry, IgM, IgG, IgA, bead-based assays, SARS-CoV-2, COVID-19 immune response

## Abstract

Background/Objectives: Although the U.S. Food and Drug Administration (FDA) has approved one antiviral treatment and authorized others for emergency use, there is no fully effective antiviral therapy for coronavirus disease 2019 (COVID-19), which is caused by the severe acute respiratory syndrome coronavirus 2 (SARS-CoV-2). Assays detecting virus-specific immunoglobulins (Ig) or nucleic acids in large-scale epidemiological, vaccine, and drug development studies remain limited due to high costs, reagent accessibility, and cumbersome protocols. Methods: A multiplex bead-based assay was developed to simultaneously detect human IgM, IgG, and IgA antibodies against the SARS-CoV-2 spike receptor binding domain (RBD) in serum using flow cytometry. Assay performance was evaluated for sensitivity, specificity, reproducibility, and cross-reactivity and compared to another immunoassay platform. Results: The assay enabled simultaneous measurement of three antibody isotypes across 624 samples within 2 h. Intra-plate coefficients of variation (CVs) ranged from 3.16 to 6.71%, and inter-plate CVs ranged from 3.33 to 5.49%, demonstrating high reproducibility. The platform also quantified background noise from nonspecific binding, facilitating straightforward data interpretation. Conclusions: This novel, flexible multiplex bead-based assay utilizing a well-established platform provides a rapid and reproducible approach for detecting SARS-CoV-2-specific antibodies. Its high throughput capacity and low variability make it well suited for large-scale epidemiological, vaccine, and therapeutic studies. The platform’s adaptability further supports application to other infectious diseases, offering an ideal tool for broad immunological surveillance.

## 1. Introduction

Coronaviruses represent a group of enveloped viruses with positive-sense single-stranded RNA with one of the largest genomes among RNA viruses, with sizes ranging from approximately 26 to 32 kilobases [1,2]. One of the characteristic features of coronaviruses is their crown-shaped spikes on the viral surface, which play a critical role in viral entry into host cells of mammals and birds [1,3,4]. In humans, three distinct coronaviruses that cause respiratory tract infections include the severe acute respiratory syndrome coronavirus (SARS-CoV), Middle East respiratory syndrome coronavirus (MERS-CoV), and severe acute respiratory syndrome coronavirus 2 (SARS-CoV-2), which were first reported in 2002, 2012, and 2019, respectively [2,5,6,7].

Coronavirus disease 2019 (COVID-19), which is caused by infection with SARS-CoV-2, was declared a worldwide pandemic by the World Health Organization in March 2020. By November 2021, over 245 million infections and 5 million deaths associated with COVID-19 were reported worldwide. Approximately 20% of COVID-19 patients are asymptomatic, while others experience a wide range of symptoms, such as fever, muscle pain, headache, sudden loss of taste, and sore throat [5,8]. Importantly, asymptomatic and pre-symptomatic individuals can still infect others [8], while advanced age and underlying conditions are associated with increased morbidity and mortality [9,10,11,12]. For example, patients who are >70 years old account for 70% of all COVID-19-related deaths in the United States. Extensive production of cytokines, which is also known as a “cytokine storm,” can perpetuate acute respiratory distress syndrome (ARDS) and organ failure in COVID-19 patients [13,14,15].

Detection of humoral antibodies to SARS-CoV-2 antigens is critical in slowing the spread of COVID-19. Routine antibody testing will help determine who has been infected with SARS-CoV-2 and which vaccinated individuals may require additional booster shots. Clinical assessment of antibodies has been used in trials that assess interventions in SARS-CoV-2 infection to improve understanding of clinical outcomes [16]. Assays that quantitatively or qualitatively determine the presence of antibodies have been used since the beginning of the pandemic in both a research-use-only setting and a diagnostic setting [17]. Currently, the antibody titers of a specific immunoglobulin (Ig) isotype for hundreds of patients can be determined with a plate-based indirect enzyme-linked immunosorbent assay (iELISA) in ~3 h. However, one major disadvantage of this platform is that its throughput capacity is limited to the following: each antibody isotype must be detected separately. Multiplex approaches can increase the throughput of antibody detection and can be used in blood [18,19,20,21] and saliva [22]. Additionally, detection of multiple Ig subtypes (IgM, IgG, and IgA) is important as the antibody kinetics vary among isotypes after infection [23] or vaccination [24].

**Figure 1:**
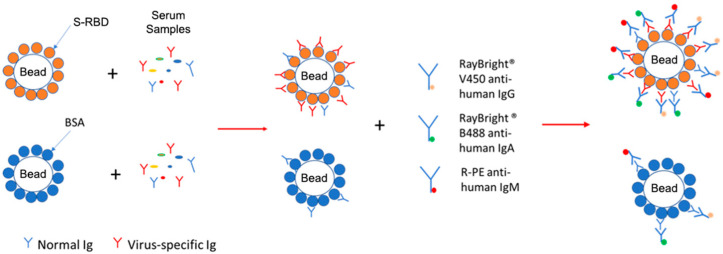
Principle of the bead-based antigen assay. Fluorescent microbeads conjugated with either SARS-CoV-2 S-RBD (antigen beads) or BSA (control beads) are incubated with serum samples, during which IgM, IgG, or IgA antibodies specific to the S-RBD will bind. Following washing to remove unbound antibodies from the beads, fluorochrome-conjugated anti-human IgM, IgG, and IgA secondary antibodies are added. Data are then analyzed with a multi-color flow cytometer.

**Figure 2:**
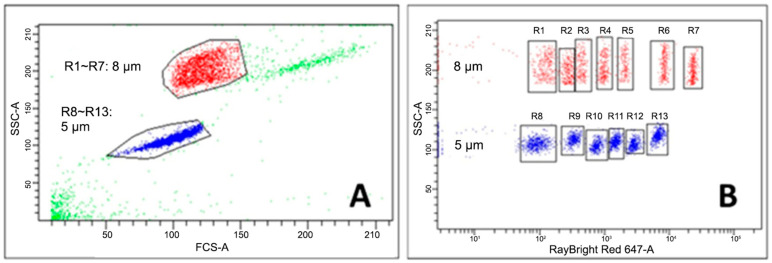
Different fluorochrome-bead size combinations enable multiplex analyses. (**A**) Beads with a 5 µm diameter occupy a distinct location within the side scatter (SSC) and forward scatter (FSC) plot compared to beads with an 8 µm diameter, thus enabling multiplex detection. (**B**) By using different APC fluorescent intensities, up to 7 samples (R1~R7) can be analyzed with the 8 µm beads, whereas up to 6 samples (R8~R13) can be analyzed with the 5 µm beads.

To overcome these limitations, we developed a high-throughput, bead-based assay for flow cytometry to detect three human Ig isotypes (IgM, IgG, and IgA) simultaneously to the SARS-CoV-2 Spike (S) protein’s receptor binding domain (RBD) within 2 h (Figure 1). To enable high-throughput analysis, 13 fluorescent beads of varying sizes (i.e., 5 and 8 µm in diameter) are barcoded with different fluorescent intensities in the allophycocyanin (APC) channel (Figure 2). Anti-human IgM, IgG, and IgA were also conjugated with three different fluorochromes (Figure 1). Using these different size-fluorescent combinations, three antibody isotypes in 624 serum samples can be analyzed simultaneously in a standard 96-well V-bottom microplate using a flow cytometer’s forward scatter/side scatter plot (FSC/SSC) and APC channel. Furthermore, antibody signal is measured accurately with control beads that account for nonspecific binding, thus making data interpretation easy. This high-throughput bead-based assay could be used for population-wide screening for COVID-19 and other infectious diseases.

## 2. Materials and Methods

**Serum Samples**. One hundred twenty-one (121) serum samples from patients confirmed to have COVID-19 via quantitative reverse transcription polymerase chain reaction (qRT-PCR) were purchased from Vitrologic, Inc. (2) (Vitrologic, Inc., Charleston, SC, USA), Cantor BioConnect Inc. (45) (Cantor BioConnect Inc., Santee, CA, USA), and Texas Direct Diagnostics (74) (Texas Direct Diagnostic, San Marcos, TX, USA). Samples were collected between March 2020 and May 2020. Of these 121 samples, the age at collection ranged from 16 to 92, with a median age of 61. 64 identified as female, 53 identified as male, and 4 did not share this information. The samples were collected between 7 days and 60 days post symptom onset and at an average of 26 days post date of PCR test (with a range of 2 days to 53 days). Two hundred ninety-nine (299) serum samples from “normal” donors were purchased from Vitrologic, Inc., before the first case of COVID-19 was reported in December 2019; thus, these donors did not have COVID-19. The age at collection ranged from 29 to 64, with a median age of 45. Among these 299 normal donors, 100 identified as female, 100 identified as male, and 99 did not share this information. Serum from patients who tested positive for antinuclear antibodies (ANA), hepatitis C, or respiratory syncytial virus (RSV), was purchased from Cantor BioConnect (Cantor BioConnect Inc., Santee, CA, USA).

**SARS-CoV-2 Antigen**. Recombinant SARS-CoV-2 spike receptor binding domain (S-RBD) with a C-terminal 6x histidine (His) tag was expressed and purified. Briefly, S-RBD (Arg319-Phe541; Accession number QHD43416) was expressed in human embryonic kidney 293 (HEK293) suspension cells via transient transfection. The protein was then purified by immobilized metal affinity chromatography (IMAC) and buffer exchanged into 1x phosphate-buffered saline (PBS), pH 7.4, with a Thermo Scientific^TM^ Slide -A-Lyzer^TM^ Dialysis Cassette, 3.5 K MWCO (Thermo Fisher Scientific; Waltham, MA, USA). Protein purity was >95% as determined via an SDS-PAGE gel stained with Coomassie blue.

**Fluorochrome-conjugated Human IgM, IgG, and IgA Detection Antibody Cocktail**. Antibodies to human IgM Fc5μ, IgG Fcγ, or IgA ɑ-chain were purchased from Jackson ImmunoResearch (Jackson ImmunoResearch Labs, West Grove, PA, USA) and conjugated with R-phycoerythrin (R-PE), RayBright^®^ Violet 450 (V450), or RayBright^®^ Blue 488 (B488), respectively. RayBright^®^ V450 and B488 were purchased from RayBiotech Life (RayBiotech Life Inc., Peachtree Corners, GA, USA), whereas the R-PE was purchased from Columbia Biosciences (Columbia Biosciences, Frederick, MD, USA). The detection antibodies were mixed to form the “Detection Antibody Cocktail.”

**Immobilization of SARS-CoV-2 S-RBD or BSA to RayPlex^®^ Multiplex Beads**. Carboxyl-coated RayPlex^®^ Multiplex Beads (RayBiotech Life, Inc., Peachtree Corners, GA, USA) were barcoded using different fluorescent intensities specific to the allophycocyanin (APC) channel and bead sizes (5 and 8 µm) (Figure 2). SARS-CoV-2 S-RBD was covalently immobilized onto each of the 13 RayPlex multiplex beads using 1-Ethyl-3-(3-dimethylaminopropyl) carbodiimide (EDC)/sulfo-N-hydroxysuccinimide (sulfo-NHS) chemistry. Briefly, 1 mg of carboxyl-conjugated RayPlex^®^ beads in 500 µL activation buffer [50 mM 2-(N-morpholino)ethanesulfonic acid (MES) buffer pH 5.0 and 0.002% Tween^®^ 20] were activated with 36 mM EDC/7.2 mM sulfo-NHS (CovaChem, LLC; Loves Park, IL, USA) for 1 hr. Activated beads were washed twice with 1 mL of coupling buffer (PBS, 0.002% Tween^®^ 20) and then incubated with 15 µg of purified SARS-CoV-2 S-RBD or bovine serum albumin (BSA) in 50 µL of coupling buffer with gentle shaking for 3 hrs. Immobilization was quenched with 50 mM Tris (pH 8.0). The beads coated with SARS-CoV-2 S-RBD are hereafter referred to as “Antigen Beads” (R1-RBD to R13-RBD), while the BSA-coated beads are referred to as “Control Beads” (R1-CTL to R13-CTL). The beads were stored in storage buffer (PBS, 0.2% *w*/*v* BSA, 0.05% *w*/*v* NaN3) at 4 °C until further use. Unless otherwise specified, all reagents were purchased from Sigma-Aldrich (Sigma-Aldrich Inc., St. Louis, MO, USA).

**Flow Cytometry Analysis of SARS-CoV-2-specific IgM, IgG, and IgA Antibodies**. The bead-based assay was carried out in 96-well Costar^®^ 3897 V-bottom microplates (Corning, Inc., Corning, NY, USA), with antigen beads (25 µL) in the first 4 rows (48 wells) and controls beads (25 µL) in the last 4 rows (48 wells).

Serum samples were first diluted 4000-fold in 1X Assay Diluent [PBS, 0.05% Tween^®^ 20, 1% BSA, 1% polyvinylpyrrolidone (PVP) + 1% casein] in a 2.2 mL deep storage plate (NEST Scientific USA, Inc., Beltsville, MD, USA). Twenty-five microliters (25 µL) of each diluted sample were added to one well of the V-bottom microplate with one of the 13 antigen beads and one well of the V-bottom microplate with the corresponding control beads, then mixed for 90 min at 1000 rpm at room temperature using an orbital shaker. Thus, a total of 50 µL per sample was employed, with 48 unique samples per plate. The beads were then washed with 200 µL of wash buffer (PBS, 0.05% Tween^®^ 20) and spun down at 1000 g for 5 min at room temperature.

With a multichannel pipette, beads in corresponding wells across 13 microplates were combined in 200 µL of wash buffer to form 1 single microplate (e.g., well A1 from 13 plates pooled together into well A1 of the final 96-well microplate), such that each final pooled well contained up to 13 different RayPlex^®^ bead combinations (Supplemental Figure S1). The plate was spun down at 1000 g for 5 min at room temperature, and the supernatant was removed.

To detect human IgM, IgG, and IgA bound to the antigen beads, the beads were incubated with 50 µL of a pre-titrated fluorochrome-conjugated detection antibody cocktail (R-PE goat anti-human IgM, RayBright^®^ V450 goat anti-human IgG, and RayBright^®^ B488 goat anti-human IgA) on an orbital shaker for 30 min at 1000 rpm and room temperature. After washing twice with 200 µL Wash Buffer, the beads were resuspended in 200 µL Wash Buffer and analyzed with a BD FACSCelesta flow cytometer (Becton, Dickinson and Company, Franklin Lakes, NJ, USA). Data were analyzed with FlowJo software, version 10 (Becton, Dickinson and Company, Ashland, OR, USA).

**Data Validation, Cutoff Value, and Positivity Definition**. The control beads were used to account for the background signal arising from non-specific binding of serological antibodies to proteins. The presence of antibodies to S-RBD, or “positivity,” was determined based on whether any of the antibody isotype data met two conditions: (1) Antigen Bead mean fluorescent intensity (MFI) to Control Bead MFI ratio > 1.5, and (2) Antigen Bead MFI—Control Bead MFI > 200. A serum sample is diagnosed as COVID-19 positive when any one of IgM, IgG, or IgA is positive.

**Data Analysis.** Using the MFI values for both antigen and control beads, the following statistical analysis was performed:

1. Assay Sensitivity, Specificity, Positive Predictive Value (PPV), and Negative Predictive Value (NPV). We tested 121 patient samples and 299 normal serum samples.
2. Assay Performance. Intra- and inter-plate CVs were used to determine the assay precision and reproducibility.
3. Class Specificity. Dithiothreitol (DTT) inactivates IgM antibodies, but not IgG antibodies. Here, IgM-, IgG-, and IgA-positive serum samples were either treated or not treated with 5 mM DTT at 37 °C for 15 min prior to running the bead-based assay to determine whether the detection of IgM antibodies was specific.
4. Cross-Reactivity. Serum from donors immunized against other infectious agents was employed to determine the cross-reactivity of antibodies to S-RBD.

## 3. Results

### 3.1. Establishment of the Bead-Based Assay

To develop the bead-based assay to detect serological IgM, IgG, and IgA antibodies to SARS-CoV-2 S-RBD, we immobilized recombinant S-RBD on 13 RayPlex^®^ multiplex beads (“Antigen Beads”). In parallel, BSA was immobilized to another set of beads (“Control Beads”). The control beads enabled the measurement of background noise arising from nonspecific antibody binding. Serum samples were incubated with antigen or control beads. To determine the optimal conditions for the assay, we compared different serum dilutions (Table 1), detection antibody dilutions, and incubation times. Optimal conditions were defined as those resulting in the highest MFI signal-to-noise (S/N) ratio with minimal background, or the antigen bead MFI to the control bead MFI following incubation with the serum sample. The highest MFI S/N ratio with minimal background was achieved when using a 4000-fold dilution of serum samples, a 90 min incubation with the antigen or control beads, 50 µL of the fluorochrome-conjugated detection antibody cocktail (1:100 dilution from 0.5 mg/mL of each detection antibody), and a 30 min incubation with the detection antibody cocktail at room temperature.

### 3.2. Determination of the Assay Sensitivity and Specificity

**Figure 3:**
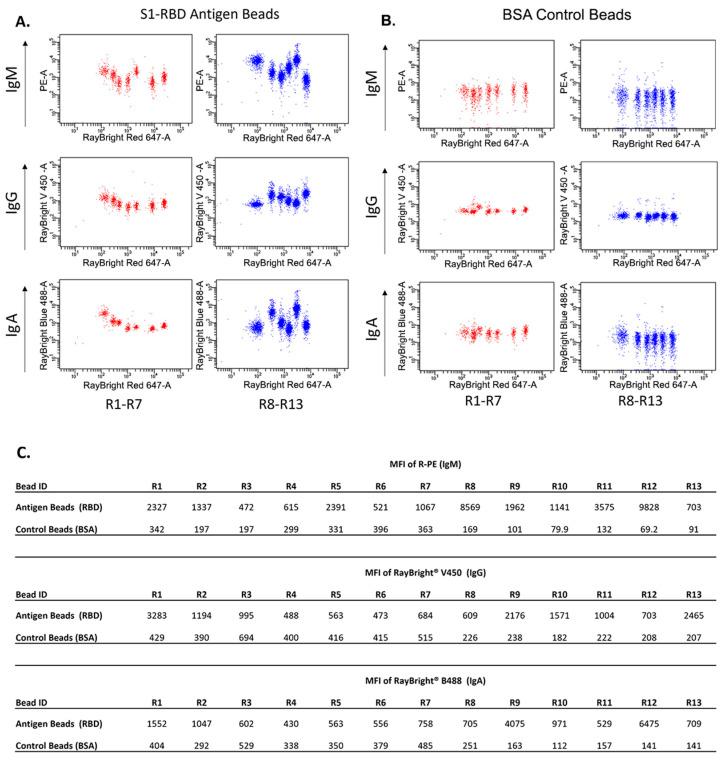
COVID-19 patient serum samples were analyzed with the bead-based assay. MFI plots of anti-human IgM, IgG, and IgA antibodies conjugated to different fluorophores for 13 COVID-19 patient sera following incubation with (**A**) antigen beads or (**B**) control beads with different fluorochrome-size combinations. Notably, the different antibody isotypes were distinguished from each other using R-PE anti-human IgM, RayBright^®^ V450 anti-human IgG, and RayBright^®^ B488 anti-human IgA. (**C**) MFI data of the antigen and control beads for 13 positive samples.

Following statistical analyses of 121 COVID-19-positive samples and 299 normal samples, the presence of antibodies to S-RBD, or “positivity,” was determined based on whether any of the antibody isotype data met two conditions: (1) Antigen Bead MFI to Control Bead MFI ratio > 1.5, and (2) Antigen Bead MFI—Control Bead MFI > 200. 119 COVID-19 positive samples and 297 negative samples were accurately classified (Supplemental Table S1), resulting in a sensitivity of 98.3% and specificity of 99.3%. A subset of these data—13 COVID-19 patient serum samples, incubated with antigen or control beads are shown in Figure 3 and Table 2 (Figure 3, Table 2). The IgM, IgG, and IgA MFI S/N ratios demonstrated that the bead-based assay accurately classified the positive and negative samples. These results suggest the bead-based high-throughput assay has very high sensitivity and specificity in identifying individuals who have been exposed to SARS-CoV-2.

### 3.3. Assay Intra- and Inter-Plate Coefficient of Variation (CV)

To evaluate the stability and reproducibility of this assay, the intra- and inter-assay CVs were determined based on the MFI S/N ratios of 13 positive samples. Intra-plate CV analysis was determined using four replicates per sample within each plate. Inter-plate CV analysis employed data across four plates. The intra- and inter-assay CVs were 3.16–6.71% and 3.33–5.49%, respectively (Table 3 and Table 4). These data demonstrate that the bead-based assay is stable and reproducible.

### 3.4. Assay Positive Predictive Value (PPV) and Negative Predictive Value (NPV) Determination

The PPV and NPV are defined as the proportion of individuals with positive or negative test results who are correctly diagnosed, respectively. Using 121 COVID-19-positive samples and 299 normal samples, the PPV was 98.3% and the NPV was 99.3%.

### 3.5. Comparison of Antibody Measurement Using ELISA

We compared the antibody detection of the multiplex bead-based assay with commercial ELISA tests that measure IgM, IgG, and IgA antibodies to SARS-CoV-2 S-RBD independently. 116 of the 121 serum samples from patients confirmed to have COVID-19 via PCR and 259 of the 299 serum samples from patients who did not have COVID-19 collected pre-2019 were analyzed. We found a 100% concordance in terms of the expected positive and negative results in the single-target ELISA compared to the multiplex array for the COVID-19 positive serum samples (98% vs. 98%, respectively). The concordance in terms of the expected positive and negative results in the single-target ELISA compared to the multiplex array for the control population was only 30% (30% vs. 99%, respectively). The IgM and IgA ELISA had a very high percentage of false positives due to high background, which was significantly improved in the multiplex array, which included a BSA control. The concordance in terms of the expected positive and negative results in the single-target ELISA compared to the multiplex array for IgG alone was the highest of the Ig isotypes (93% vs. 99%, respectively, in the control population and 89% vs. 98%, respectively, in the COVID-19-positive population). The bead-based array had increased sensitivity, specificity, and accuracy compared to the ELISA (Table 5).

### 3.6. Class Specificity

To ensure that the antibody isotype detection was specific, two patient serum samples were either treated or not treated with 5 mM DTT, since DTT is known to inactivate IgM but not IgG or IgA antibodies. Samples were then tested for IgM, IgG, and IgA antibodies to S-RBD with the bead-based assay. Both untreated samples were IgM, IgG, and IgA positive, but only IgG and IgA were positive in DTT-treated samples (Supplemental Table S1). These data indicate that antibody isotype detection with the bead-based assay is specific, particularly for IgM.

### 3.7. Crossreactivity Investigation with Samples with Non-COVID-19 Infections

Antibodies produced by the immune system in response to vaccination or previous exposure to viruses other than SARS-CoV-2 may cross-react with the S-RBD immobilized on the antigen beads, thus leading to false positives. To evaluate this possibility, 15 serum samples from patients who were clinically diagnosed with having antinuclear antibodies (ANA), hepatitis C virus (HCV), or Respiratory syncytial virus (RSV) were tested. All samples were classified as being negative for IgM, IgG, and IgA antibodies to S-RBD (Table 6).

## 4. Discussion

Large-scale screening of humoral antibodies to SARS-CoV-2 antigens is critical in epidemiological, vaccine, and drug development studies as well as slowing the spread of COVID-19 [25]. Assays that quantitatively or qualitatively determine the presence of antibodies have been used since the beginning of the pandemic in both a research-use-only as well as a diagnostic setting [16,17]. The time–course for antibody generation and detection varies, but generally IgM will become detectable 3–5 days post infection, IgA 5–7 days post infection, and IgG 7–14 days post infection. Studies have shown increased sensitivity and specificity when simultaneously detecting the different Ig subtypes [26]. Additionally, antibody testing in combination with PCR has shown improved accuracy in results [27], as together they can detect the presence of the virus or different antibody subtypes over the varying course of disease kinetics. However, the ability to perform high-throughput testing for antibodies has been limited.

The assay described here has the capability to measure 3 antibody isotypes for 624 serum samples within 2 h. In this proof-of-concept study, we demonstrate three antibody isotypes (IgM, IgG, and IgA) can be measured in 420 serum samples with our bead-based assay. We further show that the assay has low inter- and intra-CVs as well as high sensitivity and specificity. One of the key advantages of this assay compared to other bead-based assays is the use of BSA-conjugated control beads. Previous bead-based assays have relied on using the absolute MFI values [28]. However, some individuals produce high levels of antibodies that bind nonspecifically to the beads and proteins, resulting in false positives. By using control beads, we could employ an MFI S/N ratio that can account for high background caused by non-specific antibody binding.

We focused on using the assay to detect antibodies to the SARS-CoV-2 S-RBD because viral entry is facilitated by the interaction between the S-RBD and the host cell’s angiotensin-converting enzyme 2 (ACE2) receptor. As such, the S-protein and the S-RBD are major foci of vaccine and drug development efforts to prevent and treat COVID-19. However, this platform could be easily employed to study antibodies to other SARS-CoV-2 antigens.

Other non-invasive sample types include dried blood spots (DBS) and saliva. When these samples were tested with the assay, the data were inconsistent. However, further investigation using these sample types is warranted since DBS is a convenient sample type in resource-poor settings compared to serum or plasma. Furthermore, DBS can also be collected conveniently in the patient’s home and sent, via mail at room temperature, to a laboratory for testing.

High-throughput multiplex assays like the one developed here offer substantial advantages in both cost-effectiveness and labor efficiency compared to traditional single-target methods by reducing reagent consumption, sample volume requirements, and the number of assay runs needed to generate comprehensive datasets. This consolidation translates into lower per-target costs and minimizes waste. In addition, the streamlined workflow reduces hands-on time for laboratory personnel, alleviates bottlenecks associated with sample preparation and processing, and accelerates data acquisition. Collectively, these efficiencies support not only economic benefits but also faster turnaround times, making multiplex assays particularly valuable for large-scale studies, biomarker discovery, and translational research applications.

Notably, the criteria for identifying a sample as positive or negative for COVID-19 with this assay were determined using a large patient cohort but not validated using an independent cohort. Also, only a small cohort of non-COVID infectious samples were tested to determine the cross-reactivity of the assay to non-COVID conditions. Other types of infectious samples and larger cohorts should be employed in the future to validate the data. It is worth noting that while we used the assay to detect antibodies to S-RBD, other studies have shown that the immune system also responds to other SARS-CoV-2 antigens [29,30,31]. Thus, the use of other SARS-CoV-2 antigens with the assay should be explored.

## 5. Conclusions

Our bead-based multiplex assay is reproducible and high throughput, enabling simultaneous detection of IgM, IgG, and IgA antibodies to SARS-CoV-2 using standard flow cytometry. It is flexible across a wide range of applications and is compatible with standard flow cytometry, requiring only red and blue lasers. In the context of COVID-19, the assay will help slow the spread of COVID-19 by enabling routine population-wide screening of SARS-CoV-2 antibodies to help identify those who should self-quarantine and those who may require additional booster shots following COVID-19 vaccination. It offers significant advantages in efficiency, scalability, and interpretability, making it suitable for population-wide antibody screening, vaccine monitoring, and epidemiological studies. Beyond COVID-19, the platform’s adaptability supports its application to other infectious diseases, highlighting its potential as a broadly useful immunological tool.

## Figures and Tables

**Table 1:** Seven randomly chosen COVID-19 patient serum samples were serially diluted as indicated and incubated with R1~R7 beads, respectively. After washing, the beads were incubated with R-PE-conjugated goat anti-human IgM (Fc), RayBright^®^ 450-conjugated goat anti-human IgG (Fc), and RayBright^®^ 488-conjugated goat anti-human IgA (ɑ-chain) for 30 min and analyzed on a BD FACSCelesta flow cytometer. MFI readings are shown.

Sample Dilution	Beads	Sample#1			Sample#2			Sample#3			Sample#4			Sample#5			Sample#6			Sample#7		
		IgM	IgG	IgA	IgM	IgG	IgA	IgM	IgG	IgA	IgM	IgG	IgA	IgM	IgG	IgA	IgM	IgG	IgA	IgM	IgG	IgA
1/2000	Control Beads	575	1158	311	575	1035	260	554	1538	300	638	1077	314	787	1220	375	749	1216	395	745	1378	419
	Antigen Beads	20,168	59,470	12,889	20,353	55,955	12,540	15,711	51,380	10,132	22,991	65,758	14,782	25,891	70,530	16,445	23,992	63,205	14,827	22,301	53,946	13,166
	Ratio	**35.07**	**51.36**	**41.44**	**35.40**	**54.06**	**48.23**	**28.36**	**33.41**	**33.77**	**36.04**	**61.06**	**47.08**	**32.90**	**57.81**	**43.85**	**32.03**	**51.98**	**37.54**	**29.93**	**39.15**	**31.42**
1/4000	Control Beads	326	962	216	293	809	187	294	1216	187	325	900	224	423	995	249	438	989	278	438	1231	357
	Antigen Beads	10,194	32,142	6416	10,256	30,335	6436	8014	27,267	5090	11,763	35,976	7517	13,409	37,430	8337	12,464	34,474	7586	11,515	29,424	6840
	Ratio	**31.27**	**33.41**	**29.70**	**35.00**	**37.50**	**34.42**	**27.26**	**22.42**	**27.22**	**36.19**	**39.97**	**33.56**	**31.70**	**37.62**	**33.48**	**28.46**	**34.86**	**27.29**	**26.29**	**23.90**	**19.16**
1/8000	Control Beads	186	813	163	158	714	133	157	1047	147	179	775	170	231	865	194	257	849	233	325	1124	327
	Antigen Beads	5410	17,585	3436	5560	17,214	3395	4075	14,737	2588	6436	20,540	4026	7203	21,897	4425	6696	19,034	4075	6243	17,005	3765
	Ratio	**29.09**	**21.63**	**21.08**	**35.19**	**24.11**	**25.53**	**25.96**	**14.08**	**17.61**	**35.96**	**26.50**	**23.68**	**31.18**	**25.31**	**22.81**	**26.05**	**22.42**	**17.49**	**19.21**	**15.13**	**11.51**
1/16000	Control Beads	91.6	823	143	95.9	688	119	89.7	1051	123	103	765	142	132	897	150	174	854	199	254	1103	304
	Antigen Beads	2951	9798	1909	2871	9332	1801	2189	8186	1416	3468	11,001	2216	3870	11,944	2450	3619	10,477	2306	3426	9361	2150
	Ratio	**32.22**	**11.91**	**13.35**	**29.94**	**13.56**	**15.13**	**24.40**	**7.79**	**11.51**	**33.67**	**14.38**	**15.61**	**29.32**	**13.32**	**16.33**	**20.80**	**12.27**	**11.59**	**13.49**	**8.49**	**7.07**
1/32000	Control Beads	58.4	791	140	51.3	655	109	47.3	977	116	61.5	720	122	89.9	831	142	135	811	192	218	1073	300
	Antigen Beads	1524	5090	995	1528	4848	953	1180	4534	782	1807	5874	1201	2054	6281	1345	1944	5628	1246	1840	5137	1261
	Ratio	**26.10**	**6.43**	**7.11**	**29.79**	**7.40**	**8.74**	**24.95**	**4.64**	**6.74**	**29.38**	**8.16**	**9.84**	**22.85**	**7.56**	**9.47**	**14.40**	**6.94**	**6.49**	**8.44**	**4.79**	**4.20**
1/64000	Control Beads	43.2	752	126	38.4	638	105	38.6	936	104	45.6	692	121	65.9	799	139	111	789	177	200	1057	293
	Antigen Beads	784	3098	575	784	2880	566	594	2819	452	930	3468	645	1035	3800	720	1051	3374	727	1054	3204	770
	Ratio	**18.15**	**4.12**	**4.56**	**20.42**	**4.51**	**5.39**	**15.39**	**3.01**	**4.35**	**20.39**	**5.01**	**5.33**	**15.71**	**4.76**	**5.18**	**9.47**	**4.28**	**4.11**	**5.27**	**3.03**	**2.63**
1/128000	Control Beads	36.7	736	124	30.4	628	93.6	36.6	925	102	36.8	688	116	62.6	796	138	97.3	784	181	197	1064	297
	Antigen Beads	382	1835	345	398	1685	314	299	1852	276	448	2029	370	512	2196	428	494	1986	430	561	2066	507
	Ratio	**10.41**	**2.49**	**2.78**	**13.09**	**2.68**	**3.35**	**8.17**	**2.00**	**2.71**	**12.17**	**2.95**	**3.19**	**8.18**	**2.76**	**3.10**	**5.08**	**2.53**	**2.38**	**2.85**	**1.94**	**1.71**
1/256000	Control Beads	29.9	756	116	20.7	634	103	31.6	953	109	31.9	705	117	53.4	816	137	93.3	811	185	191	1064	294
	Antigen Beads	220	1296	241	223	1165	207	174	1374	199	237	1292	245	304	1464	291	302	1374	325	383	1538	406
	Ratio	**7.36**	**1.71**	**2.08**	**10.77**	**1.84**	**2.01**	**5.51**	**1.44**	**1.83**	**7.43**	**1.83**	**2.09**	**5.69**	**1.79**	**2.12**	**3.24**	**1.69**	**1.76**	**2.01**	**1.45**	**1.38**

**Table 2:** Determination of sample positivity. Exported MFI and ratio of MFI for R-PE anti-human IgM, RayBright^®^ V450 anti-human IgG, and RayBright^®^ B488 anti-human IgA of each sample using antigen beads and control beads are shown. Positivity is determined by meeting two conditions: (1) antigen bead mean fluorescent intensity (MFI) to control bead MFI ratio > 1.5, and (2) antigen bead MFI—control bead MFI > 200. A serum sample is diagnosed COVID-19 positive (+) when either one of IgM, IgG, or IgA is positive, otherwise the sample is considered COVID-19 negative (-).

**Bead ID**	**MFI of R-PE (IgM)**												
	**R1**	**R2**	**R3**	**R4**	**R5**	**R6**	**R7**	**R8**	**R9**	**R10**	**R11**	**R12**	**R13**
**Antigen Beads (RBD)**	2327	1337	472	615	2391	521	1067	8569	1962	1141	3575	9828	703
**Control Beads (BSA)**	342	197	197	299	331	396	363	169	101	79.9	132	69.2	91
**Ratio**	6.8	6.8	2.4	2.1	7.2	1.3	2.9	50.7	19.4	14.3	27.1	142.0	7.7
**Antigen Beads—Control Beads**	1985	1140	275	316	2060	125	704	8400	1861	1061	3443	9759	612
**Expected Positivity**	+	+	+	+	+	+	+	+	+	+	+	+	+
**Testing Positivity**	+	+	+	+	+	-	+	+	+	+	+	+	+
													
**Bead ID**	**MFI of RayBright^®^ V450 (IgG)**												
	**R1**	**R2**	**R3**	**R4**	**R5**	**R6**	**R7**	**R8**	**R9**	**R10**	**R11**	**R12**	**R13**
**Antigen Beads (RBD)**	3283	1194	995	488	563	473	684	609	2176	1571	1004	703	2465
**Control Beads (BSA)**	429	390	694	400	416	415	515	226	238	182	222	208	207
**Ratio**	7.7	3.1	1.4	1.2	1.4	1.1	1.3	2.7	9.1	8.6	4.5	3.4	11.9
**Antigen Beads—Control Beads**	2854	804	301	88	147	58	169	383	1938	1389	782	495	2258
**Expected Positivity**	+	+	+	+	+	+	+	+	+	+	+	+	+
**Testing Positivity**	+	+	-	-	-	-	-	+	+	+	+	+	+
													
**Bead ID**	**MFI of RayBright^®^ B488 (IgA)**												
	**R1**	**R2**	**R3**	**R4**	**R5**	**R6**	**R7**	**R8**	**R9**	**R10**	**R11**	**R12**	**R13**
**Antigen Beads (RBD)**	1552	1047	602	430	563	556	758	705	4075	971	529	6475	709
**Control Beads (BSA)**	404	292	529	338	350	379	485	251	163	112	157	141	141
**Ratio**	3.8	3.6	1.1	1.3	1.6	1.5	1.6	2.8	25.0	8.7	3.4	45.9	5.0
**Antigen Beads—Control Beads**	1148	755	73	92	213	177	273	454	3912	859	372	6334	568
**Expected Positivity**	+	+	+	+	+	+	+	+	+	+	+	+	+
**Testing Positivity**	+	+	-	-	+	-	+	+	+	+	+	+	+

**Table 3:** Intra-plate CV for IgM, IgG, and IgA detection. Representative positive samples were analyzed in quadruplicate on the same plate. Intra-plate CV% between antigen beads and control beads are shown.

Bead ID	IgM MFI						Bead ID	IgG MFI						Bead ID	IgA MFI				
	1	2	3	4	Intra-Plate CV			1	2	3	4	Intra-Plate CV			1	2	3	4	Intra-Plate CV
R1 (Antigen)	557	528	525	440	8.52%		R1 (Antigen)	484	475	480	455	2.35%		R1 (Antigen)	568	523	552	510	4.26%
R2 (Antigen)	482	445	471	403	6.75%		R2 (Antigen)	452	444	442	430	1.78%		R2 (Antigen)	489	464	482	435	4.46%
R3 (Antigen)	429	429	434	394	3.80%		R3 (Antigen)	521	539	530	528	1.21%		R3 (Antigen)	445	403	441	409	4.40%
R4 (Antigen)	544	539	510	533	2.45%		R4 (Antigen)	487	481	493	465	2.17%		R4 (Antigen)	584	570	552	526	3.89%
R5 (Antigen)	701	613	671	582	7.29%		R5 (Antigen)	529	528	522	515	1.07%		R5 (Antigen)	619	588	636	579	3.80%
R6 (Antigen)	680	642	660	615	3.68%		R6 (Antigen)	454	446	454	433	1.92%		R6 (Antigen)	643	603	637	580	4.17%
R7 (Antigen)	744	726	728	672	3.79%		R7 (Antigen)	501	509	502	486	1.68%		R7 (Antigen)	709	722	719	657	3.75%
R8 (Antigen)	219	221	226	184	7.84%		R8 (Antigen)	239	241	235	231	1.62%		R8 (Antigen)	214	208	208	190	4.39%
R9 (Antigen)	234	224	251	214	5.92%		R9 (Antigen)	264	265	259	247	2.77%		R9 (Antigen)	228	218	232	215	3.13%
R10 (Antigen)	210	215	220	182	7.12%		R10 (Antigen)	205	206	202	195	2.13%		R10 (Antigen)	201	185	194	172	5.77%
R11 (Antigen)	243	233	233	220	3.52%		R11 (Antigen)	231	234	236	223	2.14%		R11 (Antigen)	233	229	229	215	3.02%
R12 (Antigen)	193	176	195	165	6.80%		R12 (Antigen)	198	197	199	189	2.02%		R12 (Antigen)	194	187	191	165	6.18%
R13 (Antigen)	210	201	204	194	2.85%		R13 (Antigen)	226	229	222	218	1.85%		R13 (Antigen)	234	227	226	207	4.48%
R1 (Control)	228	217	230	218	2.60%		R1 (Control)	306	309	320	307	1.80%		R1 (Control)	266	258	263	250	2.34%
R2 (Control)	175	182	168	198	6.15%		R2 (Control)	279	268	288	272	2.74%		R2 (Control)	219	208	205	202	3.08%
R3 (Control)	200	203	206	187	3.64%		R3 (Control)	418	409	418	415	0.89%		R3 (Control)	234	219	222	226	2.50%
R4 (Control)	227	209	210	192	5.91%		R4 (Control)	288	282	310	287	3.69%		R4 (Control)	248	248	250	250	0.40%
R5 (Control)	259	267	275	293	4.61%		R5 (Control)	346	318	335	311	4.21%		R5 (Control)	280	264	267	266	2.34%
R6 (Control)	289	300	293	286	1.80%		R6 (Control)	285	283	281	281	0.59%		R6 (Control)	317	302	316	306	2.07%
R7 (Control)	391	407	409	391	2.14%		R7 (Control)	347	328	355	345	2.86%		R7 (Control)	419	418	403	406	1.72%
R8 (Control)	107	90	103	102	6.31%		R8 (Control)	176	169	176	171	1.78%		R8 (Control)	107	102	108	106	2.15%
R9 (Control)	93	93	102	94	3.95%		R9 (Control)	180	169	181	182	2.95%		R9 (Control)	98	104	105	101	2.68%
R10 (Control)	88	86	79	71	8.24%		R10 (Control)	140	136	141	142	1.63%		R10 (Control)	81	84	83	85	1.78%
R11 (Control)	96	109	103	111	5.58%		R11 (Control)	170	161	168	169	2.12%		R11 (Control)	119	116	115	110	2.82%
R12 (Control)	62	61	73	64	7.30%		R12 (Control)	135	127	136	134	2.66%		R12 (Control)	91	85	86	85	2.87%
R13 (Control)	77	75	86	77	5.42%		R13 (Control)	169	159	164	163	2.18%		R13 (Control)	119	114	117	117	1.53%
**Average Intra-plate MFI CV:**					**5.15%**		**Average Intra-plate MFI CV:**					**2.11%**		**Average Intra-plate MFI CV:**					**3.23%**
**Average Intra-plate MFI Ratio CV:**					**6.71%**		**Average Intra-plate MFI Ratio CV:**					**3.16%**		**Average Intra-plate MFI Ratio CV:**					**3.82%**

**Table 4:** Inter-plate CV for IgM, IgG, and IgA detection. Representative positive samples were analyzed in quadruplicate per plate. The average MFI per plate was then compared across four different plates. Intra-plate CV% between antigen beads and control beads are shown.

Bead ID	IgM MFI						Bead ID	IgG MFI						Bead ID	IgA MFI				
	1	2	3	4	Inter-Plate CV			1	2	3	4	Inter-Plate CV			1	2	3	4	Inter-Plate CV
R1 (Antigen)	2852	2886	2894	2911	0.74%		R1 (Antigen)	1333	1632	1361	1395	8.29%		R1 (Antigen)	1014	1116	1051	1036	3.60%
R2 (Antigen)	2619	2673	2689	2705	1.21%		R2 (Antigen)	1177	1338	1207	1238	4.88%		R2 (Antigen)	900	927	892	930	1.81%
R3 (Antigen)	2116	2201	2145	2226	2.01%		R3 (Antigen)	1141	1375	1158	1203	7.61%		R3 (Antigen)	761	774	783	777	1.04%
R4 (Antigen)	3209	3376	3435	3306	2.53%		R4 (Antigen)	1487	1723	1537	1553	5.64%		R4 (Antigen)	1121	1187	1205	1166	2.68%
R5 (Antigen)	3376	3567	3554	3601	2.48%		R5 (Antigen)	1504	1792	1615	1685	6.35%		R5 (Antigen)	1201	1225	1334	1243	4.02%
R6 (Antigen)	3212	3353	3320	3313	1.60%		R6 (Antigen)	1357	1556	1436	1459	4.89%		R6 (Antigen)	1161	1205	1168	1245	2.80%
R7 (Antigen)	3010	3054	3147	3171	2.13%		R7 (Antigen)	1308	1430	1342	1377	3.31%		R7 (Antigen)	1150	1202	1190	1198	1.74%
R8 (Antigen)	1019	1011	1066	1095	3.29%		R8 (Antigen)	526	561	530	562	3.09%		R8 (Antigen)	368	358	364	391	3.38%
R9 (Antigen)	1149	1140	1166	1192	1.70%		R9 (Antigen)	558	620	568	580	4.05%		R9 (Antigen)	388	403	406	411	2.13%
R10 (Antigen)	1059	1055	1093	1072	1.39%		R10 (Antigen)	456	513	485	496	4.25%		R10 (Antigen)	351	362	361	365	1.46%
R11 (Antigen)	1175	1213	1180	1213	1.49%		R11 (Antigen)	524	572	528	551	3.55%		R11 (Antigen)	388	421	396	422	3.69%
R12 (Antigen)	936	936	932	995	2.76%		R12 (Antigen)	414	471	427	437	4.83%		R12 (Antigen)	325	339	336	336	1.60%
R13 (Antigen)	1032	1079	975	1051	3.68%		R13 (Antigen)	450	535	450	478	7.26%		R13 (Antigen)	360	372	365	374	1.52%
R1 (Control)	201	210	217	183	6.28%		R1 (Control)	384	410	395	391	2.41%		R1 (Control)	266	278	259	265	2.58%
R2 (Control)	147	162	167	154	4.85%		R2 (Control)	305	336	326	316	3.59%		R2 (Control)	201	198	189	202	2.59%
R3 (Control)	183	166	173	164	4.33%		R3 (Control)	446	477	467	464	2.41%		R3 (Control)	215	226	225	217	2.18%
R4 (Control)	177	217	200	176	8.88%		R4 (Control)	350	366	363	351	1.98%		R4 (Control)	245	238	248	241	1.57%
R5 (Control)	218	263	240	255	7.02%		R5 (Control)	386	419	403	398	2.95%		R5 (Control)	270	266	254	266	2.27%
R6 (Control)	276	287	270	281	2.25%		R6 (Control)	361	384	374	365	2.39%		R6 (Control)	305	297	303	300	1.01%
R7 (Control)	375	394	372	377	2.26%		R7 (Control)	447	467	462	458	1.61%		R7 (Control)	399	421	414	437	3.27%
R8 (Control)	90	94	99	90	3.97%		R8 (Control)	209	221	211	212	2.16%		R8 (Control)	108	115	109	115	2.93%
R9 (Control)	76	92	82	99	10.17%		R9 (Control)	204	222	211	216	3.10%		R9 (Control)	108	107	112	102	3.32%
R10 (Control)	70	71	82	78	6.60%		R10 (Control)	158	173	166	164	3.24%		R10 (Control)	82	86	85	86	1.93%
R11 (Control)	92	86	91	94	3.25%		R11 (Control)	198	212	204	204	2.43%		R11 (Control)	109	121	122	111	5.01%
R12 (Control)	55	64	62	63	5.80%		R12 (Control)	156	166	160	159	2.27%		R12 (Control)	80	93	87	86	5.33%
R13 (Control)	82	74	76	85	5.60%		R13 (Control)	192	204	199	200	2.18%		R13 (Control)	131	115	130	121	5.32%
**Average Inter-plate MFI CV:**					**3.78%**		**Average Inter-plate MFI CV:**					**3.87%**		**Average Inter-plate MFI CV:**					**2.72%**
**Average Inter-plate MFI Ratio CV:**					**5.49%**		**Average Inter-plate MFI Ratio CV:**					**3.33%**		**Average Inter-plate MFI Ratio CV:**					**3.55%**

**Table 5:** Comparison of IgM, IgG, and IgA antibody measurement in the multiplex, bead-based array and the S-RBD single-target ELISA.

		IgM, IgG, IgA Multiplex Bead-based Array	Combined IgM, IgG, IgA S1-RBD ELISA	IgM S1-RBD ELISA	IgG S1-RBD ELISA	IgA S1-RBD ELISA
Positive Population	Total Samples Tested	121	116	116	116	116
	False Negative (#)	2	2	8	20	11
Control Population	Total Samples Tested	299	259	259	259	259
	False Positive (#)	2	182	109	18	126
	Sensitivity (%)	98.3%	98.0%	93.2%	88.9%	90.5%
	Specificity (%)	99.3%	30.0%	57.9%	93.0%	51.4%
	Accuracy (%)	99.0%	51.0%	51.4%	90.0%	63.0%

**Table 6:** Cross-reactivity of serum samples from patients diagnosed with ANA, HCV, or RSV to the bead-based S-RBD array.

Disease	Patient #	IgM MFI			IgG MFI			IgA MFI		
		Antigen Beads	Control Beads	Ratio	Antigen Beads	Control Beads	Ratio	Antigen Beads	Control Beads	Ratio
ANA	1	81	75	1.08	784	793	0.99	139	137	1.01
	2	77	67	1.15	716	694	1.03	116	110	1.05
	3	37	44	0.84	919	967	0.95	112	111	1.01
	4	74	67	1.10	705	729	0.97	129	132	0.98
	5	121	115	1.05	825	821	1.00	161	148	1.09
HCV	1	134	124	1.08	817	835	0.98	204	183	1.11
	2	218	212	1.03	1116	1161	0.96	356	342	1.04
	3	20	20	1.00	412	414	1.00	57	55	1.04
	4	78	68	1.15	449	441	1.02	60	57	1.05
	5	48	63	0.76	329	329	1.00	48	42	1.14
RSV	1	72	55	1.31	435	435	1.00	57	56	1.02
	2	88	82	1.07	334	345	0.97	56	59	0.95
	3	66	54	1.22	434	443	0.98	86	85	1.01
	4	95	77	1.23	189	180	1.05	74	69	1.07
	5	105	103	1.02	185	160	1.16	98	84	1.17

## Data Availability

Please address all data requests to the corresponding author.

## Supplementary Materials

The following supporting information can be downloaded at: https://www.mdpi.com/article/10.3390/vaccines13101063/s1, Figure S1: Beads in corresponding wells across < 13 microplates were combined in 200 µL of wash buffer to form 1 single microplate. Each final pooled well contained up to 13 different RayPlex fluorescent bead combinations; Figure S2: Gating Techniques and Flow cytometer settings for the multiplex bead array for human IgM, IgG, and IgA against SARS-CoV-2. Table S1: Effect of DTT treatment on IgM specificity to SARS-CoV-2 S-RBD with the bead-based assay.
