# Supplementary figures and images for: A Novel Flow Cytometry Array for High Throughput Detection of SARS-CoV-2 Antibodies
Source: Vaccines (Basel). 2025 Oct 17;13(10):1063. doi: 10.3390/vaccines13101063 (PMC12567774; doi:10.3390/vaccines13101063)

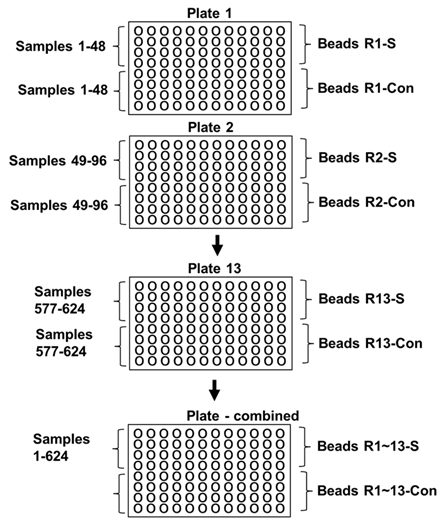

Supplement: Supplementary file 1 [file vaccines-13-01063-s001.zip › SuppFigure S1 high res.jpg]

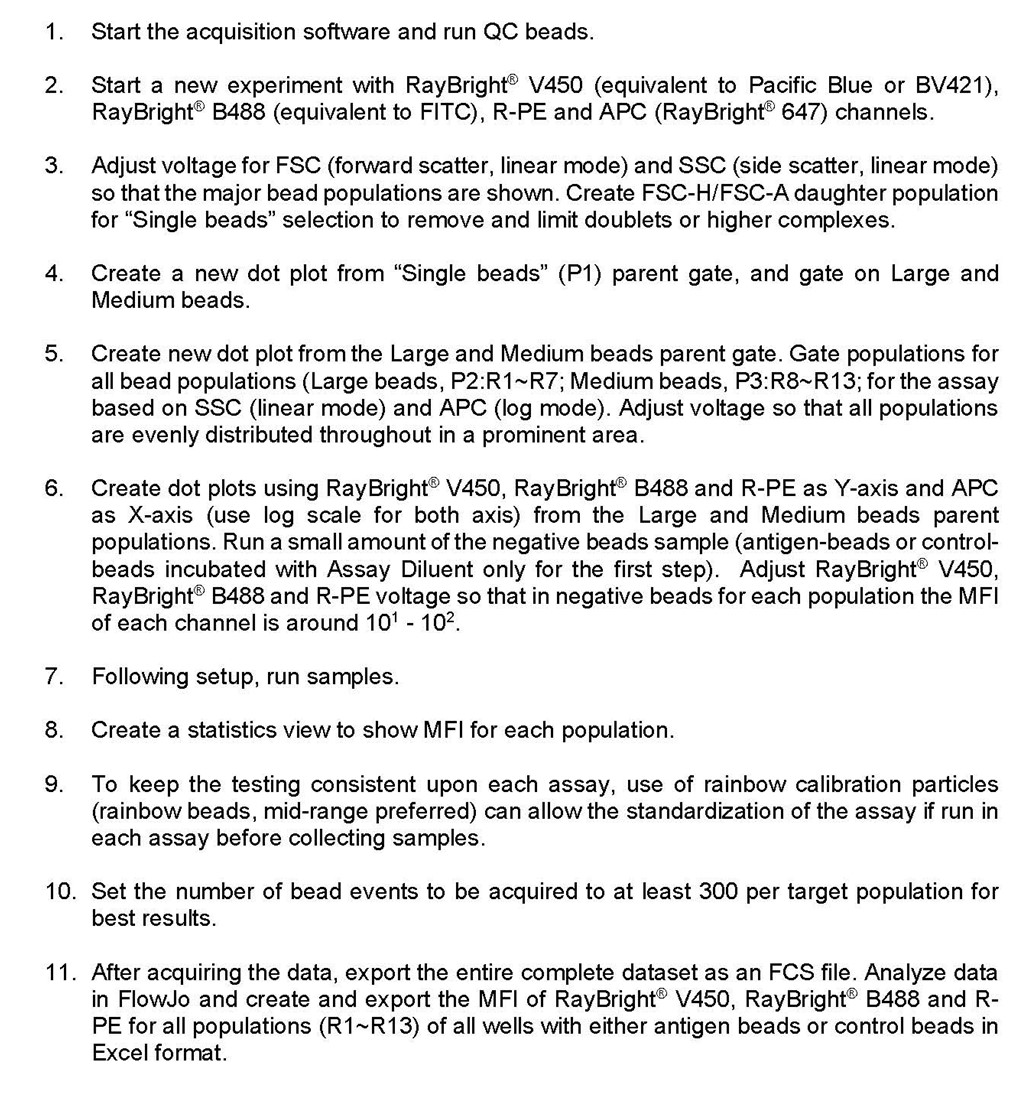

Supplement: Supplementary file 1 [file vaccines-13-01063-s001.zip › Supplemental Figure S2.jpg]

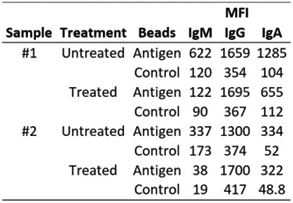

Supplement: Supplementary file 1 [file vaccines-13-01063-s001.zip › SuppTable S1 high res.jpg]
